# Methylmercury Exposure and Developmental Outcomes in Tohoku Study of Child Development at 18 Months of Age

**DOI:** 10.3390/toxics6030049

**Published:** 2018-08-21

**Authors:** Nozomi Tatsuta, Kunihiko Nakai, Mineshi Sakamoto, Katsuyuki Murata, Hiroshi Satoh

**Affiliations:** 1 Development and Environmental Medicine, Tohoku University Graduate School of Medicine, 2-1 Seiryo-machi, Aoba-ku, Sendai 980-8575, Japan; nozomi@med.tohoku.ac.jp (N.T.); sakamoto@nimd.go.jp (M.S.); 2 Environmental Health Section, Department of Environmental Science and Epidemiology, National Institute for Minamata Disease, Kumamoto 867-0008, Japan; 3 Department of Environmental Health Sciences, Akita University Graduate School of Medicine, Akita 010-8502, Japan; winestem@med.akita-u.ac.jp; 4 Environmental Health Science, Tohoku University Graduate School of Medicine, Sendai 980-8575, Japan

**Keywords:** methylmercury, prenatal exposure, child development

## Abstract

Seafood is an important component in a healthy diet and may contain methylmercury or other contaminants. It is important to recognize the risks and benefits of consuming seafood. A longitudinal prospective birth cohort study has been conducted to clarify the effects of neurotoxicants on child development—the Tohoku Study of Child Development (TSCD) in Japan. TSCD comprises two cohorts; a polychlorinated biphenyls (PCB) cohort (urban area) and a methylmercury cohort (coastal area). Our previous results from the coastal area showed prenatal methylmercury exposure affected psychomotor development in 18-month-olds, and boys appear to be more vulnerable to the exposure than girls. In this report, we have added the urban area cohort and we reanalyzed the impact of prenatal exposure to methylmercury, which gave the same results as before. These findings suggest prenatal exposure to low levels methylmercury may have adverse effects on child development, especially in boys.

## 1. Introduction

Seafood is a good source of protein and various essential nutrients including n-3 polyunsaturated fatty acids (n-3 PUFAs), selenium, iodine, and vitamin D, whereas it is low in fatty acids. Among the nutrients, n-3 PUFAs, such as docosahexaenoic acid (DHA), are important because these fats are difficult to get in sufficient amounts from other food items but are highly beneficial for maternal and fetal health because of their critical role in proper brain development and function [1,2]. However, seafood also contains several toxic chemicals like methylmercury and polychlorinated biphenyls (PCB) due to bioaccumulation in the aquatic food chain [3,4]. Human exposure to methylmercury and PCB occurs mainly from the intake of seafood [5]. Indeed, mercury exposure level is related to the amount of seafood intake [6], and several studies have confirmed a relationship between seafood intake and mercury blood/hair levels in humans [6,7,8]. Importantly, because methylmercury readily crosses the placenta, fetuses are at a high risk to exposure [9]. Many previous studies found an association between prenatal exposure to methylmercury and adverse effects on child neurodevelopment [10,11,12,13,14,15,16,17].

Because the Japanese are one of the world’s largest seafood consumers, we initiated the Tohoku Study of Child Development (TSCD) in 2001 [18]. TSCD is a prospective birth cohort study that investigates the effects of neurotoxicants on child development in Japan with the objective to determine the potential risks and benefits of seafood eating during pregnancy. TSCD consists of two birth cohorts, an urban area and a coastal area cohort, in northeastern Japan [19,20,21,22,23,24]. The purpose of the urban area cohort is to examine the effect of PCB exposure, and that of the coastal area cohort is to examine the effect of prenatal methylmercury exposure on child development. The chemical substances being measured are different, but the protocols are almost the same. We previously reported that the psychomotor development in 18-month-old children of the coastal area was significantly correlated with cord-blood total mercury (THg) only in the boys, and that this association remained significant after adjustment for possible confounders, including maternal-plasma DHA and cord-plasma selenium [22]. This result suggests that boys are more vulnerable to methylmercury exposure than girls, which is consistent with a few other studies [22,23,25,26,27]; although most other studies on this subject did not include gender-specific analyses [28]. Even if the gender was considered, most birth cohort studies in children included it as a confounding variable, which needed to be controlled and generalized the chemotoxic effects without accounting for possible gender discrepancies [29].

In this report, we introduce the exposure status of methylmercury in Japanese pregnant women, and the protocol of TSCD. Then, we also measured methylmercury exposure levels in an urban area cohort, therefore, we investigated the impact of prenatal methylmercury exposure on the developmental scores using the Bayley scales of infant development second edition (BSID-II) [30] in the urban and coastal areas for 18-month-old children, with an emphasis on the potential impact of the child gender.

## 2. Materials and Methods

### 2.1. TSCD Outline

The TSCD protocols have been described in a previous report [18]. TSCD comprises two birth cohorts from northeastern Japan, an urban area and a coastal area cohort. The first birth cohort study had been conducted in an urban area to examine the effects of perinatal low-level PCB exposure on child developmental outcomes. The second birth cohort study is being conducted to examine the effects of prenatal methylmercury exposure to child development outcomes. To decide the research field, we investigated an area with a high methylmercury exposure level. We conducted a preliminary survey to select the research area from four candidate areas in the Tohoku region. To identify the area with the highest hair THg concentration, we recruited women who visited their regional obstetrical-gynecological clinics to provide their hair samples for THg analysis. Written informed consent was obtained from each participant prior to collecting the hair samples. In addition, we referred to a study by Yasutake et al. [31], who reported hair THg concentration data from five districts in Japan. Based on all these results, we had finally decided to conduct the coastal area for our research (Candidate area A of Table 1).

To establish an optimal study population, the eligibility criteria included a singleton pregnancy, Japanese as the mother tongue, and neonates born at term (36–42 weeks of gestation) with a birth weight of more than 2400 g and no congenital anomalies or diseases. We referred to the Dutch PCB/Dioxin study before starting our cohort study [32]. One of the inclusion criteria of the Dutch study is child born at term (37–42 weeks). This is because the reason for preterm birth is thought to be affected by factors other than prenatal PCB exposure. Therefore, in our study, low birth weight children and preterm birth children were excluded. Outcome measurements of age-appropriate neurobehavioral assessments and parent report questionnaires were selected to examine the effect of prenatal methylmercury exposure. The details of the follow-up neurobehavioral assessments and questionnaires are shown in Table 2. The study protocol was approved by the Medical Ethics Committee of the Tohoku University Graduate School of Medicine. 

#### 2.1.1. Urban Area Cohort (PCB Cohort)

**Figure 1:**
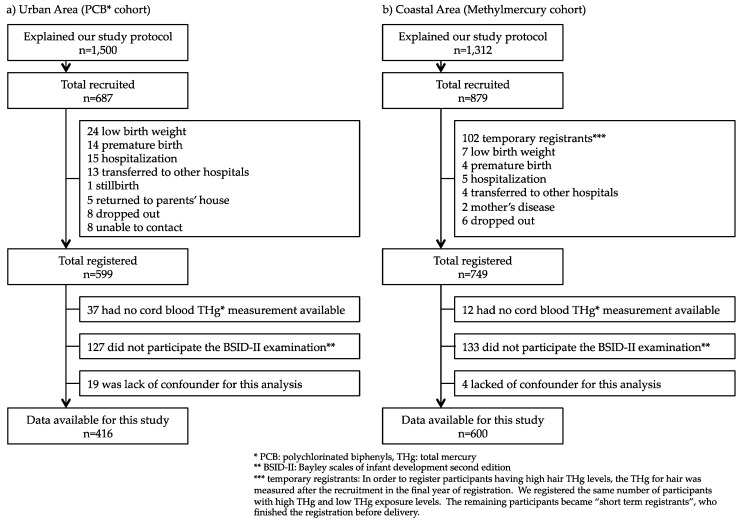
Flowchart of study population. Flowchart indicating the number of mother–child pairs included and excluded from the study population.

For the urban area cohort, we recruited 1500 of 22-week-pregnant women from January 2001 to September 2003. Of these, 687 pregnant women agreed to participate (participation rate, 45.8%) and provided written informed consent to provisionally register for this study. After birth, 88 provisional registrants withdrew from the study due to various reasons in accordance with the eligibility criteria, for example, premature birth, and being transferred to another hospital. A total of 599 mother–child pairs were finally registered. The flowchart of how the study participants were determined is shown in Figure 1a.

#### 2.1.2. Coastal Area Cohort (Methylmercury Cohort)

For the coastal area cohort, we recruited 1312 of 22-week-pregnant women from December 2002 to March 2006; 879 pregnant women agreed to participate (participation rate, 67.0%) and provided written informed consent to provisionally register for this study. While the initial registration procedure was the same as that for the urban area cohort, a screening step for identifying participants with high hair THg levels was added. Immediately after the recruitment, hair samples were obtained and analyzed for THg content. Potential participants with a high THg level were registered together with a matching number of participants with a low THg level. These low THg participants were selected with adjusting the age, sex, amount of seafood consumption, and the tendency to consume certain fish species. At this step, 102 participants were excluded from the study, because participants had lower hair THg. Finally, 749 mother–child pairs were registered. The flowchart of how the study participants were determined is shown in Figure 1b.

### 2.2. Exposure Markers

THg concentrations in whole cord blood, hair, and breast milk were analyzed using cold vapor atomic absorption spectrometry [19,20]. The analytical method for THg has been described elsewhere [20,33]. Cord blood and breast milk PCB were analyzed by high-resolution gas chromatography and high-resolution mass spectrometry using the isotope dilution method. Laboratory analytical methods and quality control procedures were described elsewhere [19,34]. Cord blood lead concentrations in whole cord blood were determined by inductively coupled plasma mass spectrometry (ICP-MS, SRL Inc., Tokyo, Japan) [19,22,23]. Cord blood selenium was analyzed by ICP-MS, and cord plasma selenium was analyzed by Watkinson method [22,23]. Maternal plasma DHA was analyzed by using gas chromatography [22].

### 2.3. Outcome

The BSID-II score was one of the study outcomes used for 18-month-old children. The raw scores were standardized for the child’s age in days at the time of test administration. The raw scores of each scale were converted into the mental developmental index (MDI) and psychomotor developmental index (PDI), based on age-appropriate norms. These scores are derived from the total raw scores of each test and normalized on a score scale with a mean of 100 and a standard deviation (SD) of 15. Since there was no standardized version of the BSID-II for Japan, we prepared a Japanese version ourselves. The reliability of administration of the BSID-II has been described elsewhere [35].

### 2.4. Confounding Variables

Information about pregnancy, delivery, and the characteristics of infants at birth such as child gender, birth weight, and birth order were obtained from medical records. We obtained information about demographics and smoking/drinking habits during pregnancy (presence/absence) from a questionnaire four days after delivery.

Maternal seafood intake during pregnancy was assessed using a food frequency questionnaire (FFQ) that was administered by trained interviewers immediately after delivery. Methylmercury intake was estimated from seafood intake and methylmercury concentrations in each type of seafood. The calculation method has been described elsewhere [36]. 

The maternal intellectual ability was evaluated using the Raven standard progressive matrices [37]. We used the raw score for analysis because it has not been standardized in Japan. Home environment was assessed using the Evaluation of Environmental Stimulation (EES) questionnaire [38], which had been established in Japan modified after home observation for measurement of the environment score [39]. The mother was asked to fill out the Raven standard progressive matrices and the EES when her child was 18 months of age.

### 2.5. Statistics

The THg in cord blood and maternal hair were logarithmically transformed (log_10_) because of skewed distributions. Sex differences in basal characteristics, exposure levels, and scores of the BSID-II were analyzed using the Student *t*-test, Mann–Whitney *U* test, or Fisher exact test. Multiple regression analysis was used to adjust for possible confounders. Independent variables in the analysis were child gender, birth weight, birth order, drinking and smoking habits, the Raven scores, EES score, and testers of the BSID-II (for which dummy variables were used). All analyses using two-sided *p*-values, were performed using SPSS Ver. 23.0 (SPSS Inc., Tokyo, Japan, 2016) and the statistical significance was set at *p* < 0.05.

## 3. Results

In the urban area cohort (PCB cohort) with 599 registered mothers, cord blood samples were collected from 562 participants (93.8%), maternal hair samples at parturition were collected from 595 participants (99.3%), and the FFQ was administered to 598 participants (99.9%). In the coastal area cohort (methylmercury cohort) with 749 registered mothers, cord blood samples were collected from 731 participants (97.6%), maternal hair samples at parturition were collected from 748 participants (99.9%), and the FFQ was administered to all participants (100.0%). In Japan, the tolerable weekly intake (TWI) for methylmercury of 2.0 μg/kg body weight per week for pregnant and potentially pregnant women was proposed by the Japan Food Safety Commission [40]. However, 12.4% of the urban area participants and 18.2% of the coastal area participants exceeded the TWI [22,36].

In this study, we excluded children who did not have cord blood samples for methylmercury biomarker, did not participate in the 18-months BSID-II examination, or lacked a confounder for this analysis. Thus, for the final analysis, 416 mother–child pairs from the urban area and 600 mother–child pairs from coastal area were included (Figure 1). The mean age of the participating children was 18 months (range, 17 to 24 months). Table 3 shows the basal characteristics of the mother–child pairs. By comparing the urban and coastal area cohorts, differences were found in many variables, such as maternal age at parturition, BMI before pregnancy, and drinking/smoking habits during pregnancy. The distributions of the biomarkers are shown in Table 4. Although the methylmercury biomarkers were significantly higher in coastal area participants than in urban ones, there was no difference in total seafood intake during pregnancy. Since the amount and species of seafood consumed were expected to be different among the districts in Japan, we examined the differences in intake of each kind of seafood (Table 5). Coastal area participants consumed more bonito, salmon, shellfish, some other kind of fish, and shellfish than the urban area participants, who consumed more whale, yellowtail, and eel. The type of seafood eaten by coastal area participants had high concentrations of methylmercury such as bonito.

The participation rate of each examination is shown in Table 6. The urban area cohort was closed after the examination at the age of 84 months due to the lack of research funds. However, while we were examining the 84-month-old children of the coastal area cohort, the Great East Japan Earthquake hit, causing severe damage. Because of this disaster, the participants were compulsorily divided into predisaster and postdisaster groups [21]. The follow-up rate for the 84-months-old examination of the predisaster group was 78.1%, but for the postdisaster group, it was 65.6%. Therefore, the follow-up rate for the 84-months-old examination was lower than the other examination. 

The scores of the BSID-II are provided in Table 7. The MDI of the BSID-II was significantly higher in the urban area children than that in the coastal area children, whereas there was no difference in the PDI of the BSID-II between these two groups. Table 8 shows the results of the multiple regression analysis. Cord-blood THg was not significantly correlated with any BSID-II scores. However, there was a relationship between child gender and BSID-II scores. Therefore, the following analysis was carried out separately for boys and girls. As shown in Table 9, it was only in boys that the cord-blood THg was significantly associated with lower PDI of the BSID-II. As we focused on the PDI score of BSID-II and stratified the participants by research area, the association between cord-blood THg and PDI was found only in the coastal area boy group (Table 10).

## 4. Discussion

### 4.1. Outline of the TSCD

In this report, we summarized the protocol and state of progress of the TSCD. The TSCD consists of participants in an urban area and coastal area, and both areas are in northeastern Japan; various differences were found in the basal characteristics as in Table 3. When clarifying the health effects of prenatal methylmercury exposure, the importance of collecting information of basal characteristics and confounding factors was shown. Although there was no difference in fish intake between the two cohorts, hair/cord-blood THg was significantly higher in the coastal participants. For this reason, it was shown that the coastal participants consume fish containing higher mercury levels than urban participants. Most fish samples contain methylmercury, but the concentrations vary greatly according to the fish species. In fact, the coastal participants consume seafood with higher methylmercury concentration such as bonito (Table 5).

In our cohort, follow-up rates of the coastal area have decreased to 69.2% in 84-month-old examination and 55.0% in 144-month-old examination, respectively as in Table 6. A possible reason why the follow-up rate of 84-month-old examination was decreased in the coastal area would be the effects of the Great East Japan Earthquake. The disaster also had several significant impacts on our participants and cohort. For instance, hair THg of the children was decreased by approximately 30% after the disaster. In these areas, the consumption of seafood was decreased after the disaster because of destructive damage to the fishery [24]. The disaster induced subtle deficits in verbal intelligence quotient (IQ) of 84-month-old children, probably due to a temporal blank in their education [21]. However, the follow-up rate improved at next examination at 120-month-old. In the 144-month-old examination follow-up rate was down to 55% as in Table 6. Reasons for not being able to participate in the examination include children being busy with study and sports club activities, and parents working, so their schedules do not match. Other than that, we thought that we were able to maintain a function as a cohort.

### 4.2. Exposure Levels

The median THg levels of maternal hair at parturition were 2.0 μg/g and 2.5 μg/g in the urban and coastal areas, respectively, which were lower than the levels reported in other studies in the Faroe Islands [41,42], the Seychelles [43], Canada [44], Brazil [6,45], and France [46], but higher than those in Italy [47], the Philippines [48], China [49], Austria [50], and the United States (US) [51]. These suggest that the level of exposure to methylmercury in our study participants was definitely not high compared with other studies. However, 93.6% of the urban area participants and 94.5% of the coastal area participants of our cohort had hair THg concentrations higher than the US Environmental Protection Agency (EPA) recommended levels (1.0 μg/g for hair THg) for optimal health. Yasutake et al. [31] reported that it would not be adequate to employ the reference dose (RfD) of the US EPA in Japan. We think that it is necessary to examine the effect of prenatal methylmercury on Japanese.

As described in the Results section, the Japan Food Safety Commission proposed TWI for THg at 2.0 μg/BW-kg/week in 2005. To apply the TWI for THg, 12.4% of pregnant women in the urban area and 18.2% of pregnant women in the coastal area exceeded it [22,36]. In Japan, the Ministry of Health, Labour, and Welfare advises that pregnant women limit consumption of certain species. However, according to the results of another of our studies, 25.5% (14/55) of women who have never been pregnant did not know about this advice, and even 47.7% (336/705) of women who had been pregnant did not know about it.

Since fish are rich in n-3 PUFAs, fish consumption is the primary source of n-3 PUFAs intake, and Saito et al. [52] reported the determinants of n-3 PUFAs status in maternal and cord blood were examined, and maternal seafood consumption was a potent factor. To avoid the adverse health effects of prenatal methylmercury while retaining the benefits provided by fish consumption, it is important to select suitable fish species and to pay attention to the amount of fish intake. For that purpose, the information and knowledge about seafood intake, including advice from the Ministry of Health, Labour, and Welfare, must be known thoroughly by pregnant women and women who may become pregnant. It is necessary to transmit correct information to next generation who become parents.

### 4.3. Gender-Specific Analyses

Compared to studies on other heavy metals (cadmium, manganese, and arsenic), studies on methylmercury and lead that were analyzed by based on child gender are more abundant. However, child gender-specific susceptibility to prenatal methylmercury exposure has not received sufficient attention [28]. When we look at just prenatal exposure to methylmercury, boys seem more susceptible to adverse effects than girls. Indeed, studies of human prenatal methylmercury poisoning in Iraq found that male newborns suffered worse complications from methylmercury exposure than female newborns [53]. In Minamata, Japan, it was reported that the male/female birth ratio decreased in the overall city population as well as in the fishing villages during 1955–1959, when the pollution degree of methylmercury was very high. Additionally, a decrease in maternal Minamata disease patients was observed, but this was due to the high number of stillborn male fetuses [26]. The adverse effects of methylmercury on child neurodevelopment were reported by cohort studies in northern Quebec [54], Seychelles [25,55], Faroe Islands [56,57], Massachusetts [58], Guiana [47], and the city of Zhoushan [50]. On the other hand, two studies reported the negative impact on girls [59,60]. According to our previous studies, the birth weight and psychomotor development of the boys were affected by cord-blood THg level [23]. The mechanism underlying gender differences in exposure related neurotoxicity is unknown, and information regarding gender differences in susceptibility of methylmercury is still too limited to draw any definite conclusions.

Understanding gender differences is useful for elucidating the pathways from exposure to manifestation, and may also provide new insights into prevention strategies [28]. In order to clarify this, further studies are required to clarify the mechanism of effects of prenatal exposure on gender difference in experimental studies. Also, sample size is an important factor that must be considered carefully. There are several large prospective birth cohort studies on environmental contaminants and child health, such as the Danish National Birth Cohort in Denmark [61], the Norwegian Mother and Child Cohort Study in Norway [62], the Newborns and Genotoxic exposure risks project in EU [63], and the Japan Environment and Children’s Study in Japan [64]. Each of these studies is made up of more than 100,000 parent–child pairs. Such large cohorts may solve this issue in the near future.

### 4.4. Regional Difference

In the current study, we found that increasing cord blood THg was associated with lower PDI of the BSID-II at 18 months of age in the coastal area boys but not in the urban area boys. We can postulate several reasons for this discrepancy. One of these reasons might be that the coastal area pregnant women in our study had a higher and wider range of cord-blood and hair THg than the urban area pregnant women. The maternal hair THg at parturition in the coastal area participants of this study ranged from 0.31 μg/g to 11.0 μg/g and in the urban area participants from 0.29 μg/g to 9.34 μg/g, respectively. The extremely low and narrow range of methylmercury concentration in the urban area participants of the present study may be the reason why we obtained insignificant results. Exposure level and range of the study population may be important for detecting a significant result.

As confounders for the BSID-II scores, birth weight, score of the maternal Raven standard progressive matrices, and score of the EES were chosen in multiple regression analysis (Table 8, Table 9 and Table 10), and there were significant differences in them between the urban area cohort and the coastal area cohort (Table 3). It is common knowledge that these variables are closely related to child development. For instance, a consistent association between birth weight and child development has been established [65]. The Port Pirie Cohort Study reported that the higher the occupational prestige and maternal IQ and the better the home environment, the higher the children’s cognitive function [66]. To clarify the effect of prenatal methylmercury exposure, these crucial confounders should be collected properly in future studies.

### 4.5. Future Vision

The Faroe cohort study showed that prenatal methylmercury related neuropsychological dysfunctions were most pronounced in the domains of language, attention, and memory, and to a lesser extent in visuospatial and motor functions at 7 and 14 years [10,40,67]. After that, they examined whether negative associations are still detectable eight years later, at age 22. The results at age 22 suggest that cognitive deficits associated with prenatal methylmercury exposure remain through young adulthood. They concluded that prenatal exposure to this marine contaminant appears to cause permanent adverse effects on cognition [68]. At the present stage, even in our cohort, the negative effect of prenatal methylmercury exposure has been observed, follow-up data are needed to determine if adverse effects occur at older ages and if such effects are determined to be related to methylmercury. We have collected data on the development at different age of our children so we are planning to analyze the effect of prenatal exposure to methylmercury on child development. As our participants are turning 12-year-old, 10 years later, we would like to reconfirm Faroe study’s results in Japan.

We are also measuring toxic chemicals other than THg, for instance PCB and lead in cord-blood and blood as in Table 4, due to estimation of the combined risks of the mixture effects is required. Exposure to a mixture of chemicals is ubiquitous in real life and all children are exposed to multiple toxic chemicals [69]. Recently, gradually the number of such studies is increasing [19,70,71,72,73,74], however, the results pattern was not so clear. A research model with concomitant exposures is necessary for evaluating subtle effects on child development. In addition, n-3 PUFAs is essential for normal brain development. Three studies reported that the negative association between prenatal methylmercury exposure and cognitive development became apparent only after adjustment with n-3 PUFAs intake [75,76,77]. These studies indicated the essential nutrients such as n-3 PUFAs masked the effects of methylmercury. Future studies need to indicate the exposure levels of both beneficial nutrients and toxic substances such as methylmercury and PCB. Moreover, we are planning to identify high-risk groups with genetic susceptibilities to methylmercury.

## 5. Conclusions

The TSCD examined both the potential risks and benefits of eating fish during pregnancy. Our participants are turning 12-year-old, and till now, we thought that we were able to maintain a function as a cohort. Results of the TSCD suggest that even relatively low levels of exposure to methylmercury may have adverse effects on child development especially in boys. Thus, a long-term follow-up study should be conducted to obtain further information about the effects of methylmercury exposure on child development.

In Japan, the TWI for methylmercury of 2.0 μg/kg body weight per week for pregnant and potentially pregnant women was decided by the Japan Food Safety Commission (2005). 12.4% of the urban area participants and 18.2% of the coastal area participants exceeded the TWI [22,36]. However, a lot of Japanese people do not know about the TWI. Seafood is an important part of a healthy diet and contains good nutritional properties for pregnant women and fetuses. Therefore, we need to present correct information about how to eat seafood. It is necessary to transmit correct information to next generation who become parents. We want to clarify an information providing method of more efficiently and effectively providing required information to pregnant women to protect our children’s lives and futures.

## Figures and Tables

**Table 1:** Geometric mean and min/max of hair THg * in candidate areas of our research area (μg/g).

Candidate Area	n	Geometric Mean	Min	Max
Candidate area A	100	3.27	1.00	13.3
Candidate area B	94	1.99	0.66	10.3
Candidate area C	99	1.80	0.55	5.35
Candidate area D	100	2.01	0.67	8.15
Yasutake et al. *Tohoku J. Exp. Med.* **2003**, *199(3)*, 161–169				
Minamata	594	1.23	0.09	7.33
Kumamoto	327	1.33	0.14	6.20
Tottori	209	1.40	0.26	12.5
Wakayama	303	1.40	0.00	8.09
Chiba	233	2.30	0.14	25.8

* Total mercury.

**Table 2:** Follow-up and outcome measures of TSCD *.

Child Age	Neurobehavioral Development Assessment
3 days	Neonatal Behavioral Assessment Scale
7 months	Kyoto Scale of Psychological Development (KSPD)
	Bayley Scales of Infant Development second edition (BSID-II)
	Fagan Test of Infant Intelligence
18 months (1.5 years)	KSPD, BSID-II, Evaluation of Environmental Stimulation (EES)
	Raven standard progressive matrices
30 months (2.5 years)	Child Behavior Checklist age for 2–3, EES
42 months (3.5 years)	Kaufman Assessment Battery for Children
66 months (5.5 years)	Social-Maturity Skill Scale (S-M scale)
84 months (7 years)	Wechsler Intelligence Scale for Children Third edition
120 months (10 years)	S-M scale
144 months (12 years)	Wechsler Intelligence Scale for Children Forth edition

* Tohoku Study of Child Development.

**Table 3:** Background characteristics of TSCD * mother–child pairs.

Basal Characterisctics	Urban Area	Coastal Area	*p-*Value **
	Mean ± SD *	Mean ± SD *	
	(or %)	(or %)	
Maternal characteristics			
Maternal age at parturition (years)	31.3 ± 4.4	29.5 ± 4.9	*p* < 0.001
Body mass index before pregnancy (kg/m^2^)	21.0 ± 2.8	21.5 ± 3.3	0.002
Drinkers during pregnancy (%, yes)	31.7	16.6	*p* < 0.001
Smokers during pregnancy (%, yes)	7.8	12.5	*p* < 0.001
Maternal education level (%, >12 y)	74.9	41.5	*p* < 0.001
Raven score ***	51.5 ± 6.3	49.9 ± 6.0	*p* < 0.001
EES score at 18 months ***	28.2 ± 3.4	26.6 ± 3.8	*p* < 0.001
Child characteristics			
Child gender (%, boys)	52.6	50.9	0.547
Birth order (%, first child)	51.4	42.1	0.001
Gestational duration (weeks)	39.5 ± 1.3	39.7 ± 1.2	0.038
Birth weight (g)	3073 ± 338	3141 ± 365	*p* < 0.001
Delivery type (%, vaginal delivery)	83.6	84.4	0.765
Apgar score (1 min)	8.2 ± 0.8	8.4 ± 0.8	*p* < 0.001

* TSCD, Tohoku Study of Child Development; SD, standard deviation. ** Student *t*-test or *χ*^2^ test. *** Raven, Raven standard progressive matrices; EES, Evaluation of Environmental Stimulation.

**Table 4:** Exposures indices in urban and coastal area participants.

Exposures	Urban Area	Coastal Area	*p-*Value *
	n, Median, 5–95 Percentiles	n, Median, 5–95 Percentiles	
Exposure biomarkers:			
Cord-blood THg (ng/g) **	562, 10.0, 4.2–22.4	731, 16.0, 5.6–39.3	*p* < 0.001
Maternal hair THg (μg/g) **	595, 2.0, 0.9–4.4	748, 2.6, 0.9–6.0	*p* < 0.001
Breast milk THg (ng/g) **	-	27, 0.8, 0.1–1.8	-
Cord-blood PCB (ng/g-lipid) **	518, 45.8, 18.4–112.2	-	-
Breast milk PCB (ng/g-lipid) **	544, 93.1, 42.4–185.9	-	-
Cord-blood lead (ng/dL)	555, 1.0, 0.6–1.8	664, 0.7, 0.4–1.4	*p* < 0.001
Cord-blood selenium (ng/mL)	555, 192.7 (130.3–271.9)	-	-
Cord-plasma selenium (ng/g)	-	709, 66.3, 51.0–271.9	-
Maternal-plasma DHA **	-	742, 169.7, 101.1–256.9	-
Seafood intake during pregnancy (kg/y)	598, 44.4, 12.6–110.8	749, 47.7, 10.5–140.6	0.089

* Mann–Whitney *U* test. ** THg, total mercury; PCB, polychlorinated biphenyls; DHA, docosahexaenoic acid.

**Table 5:** Comparison of the amount of intake of seafood determined by the FFQ * (g/day).

Fish Species	Urban Area (*n* = 598)	Coastal Area (*n* = 749)	*p-*Value **
	Median (Min-Max)	Median (Min-Max)	
Tuna	4.1 (0.0–123.7)	4.4 (0.0–105.0)	0.161
Bonito	2.1 (0.0–41.8)	2.7 (0.0–108.3)	*p* < 0.001
Whale	0.0 (0.0–17.5)	0.0 (0.0–2.3)	0.004
Salmon	3.1 (0.0–34.7)	3.1 (0.0–92.5)	0.037
Eel	0.3 (0.0–32.1)	0.0 (0.0–12.5)	*p* < 0.001
Yellowtail	0.6 (0.0–70.0)	0.0) (0.0–70.0)	*p* < 0.001
Silvery blue fish	5.8 (0.0–55.0)	5.8 (0.0–70.0)	0.546
White-meat fish	7.2 (0.0–87.0)	7.2 (0.0–87.0)	0.918
Other fish	0.0 (0.0–57.9)	3.0 (0.0–90.0)	*p* < 0.001
Squid/Octopus	2.0 (0.0–30.0)	2.0 (0.0–60.0)	0.264
Shellfish	1.7 (0.0–39.3)	2.3 (0.0–50.0)	*p* < 0.001
Salmon roe	0.0 (0.0–37.5)	0.0 (0.0–37.5)	0.162
Canned tuna	1.7 (0.0–60.0)	2.0 (0.0–60.0)	0.524

* FFQ, food frequency questionnaire. ** Mann–Whitney *U* test.

**Table 6:** Follow-up rates of each examination.

Time of Each Examination	Urban Area			Coastal Area		
	Registrants	Participants	%	Registrants	Participants	%
3 days	599	587	98.0	749	709	94.7
7 months	594	516	86.9	749	653	87.2
18 months (1.5 years)	589	477	81.0	747	617	82.6
30 months (2.5 years)	595	499	83.9	739	649	87.8
42 months (3.5 years)	566	472	83.4	733	597	81.3
66 months (5.5 years)	580	456	78.6	727	614	84.5
84 months (7 years)	546	457	83.7	720	498	69.2
120 months (10 years)				711	569	80.0
144 months (12 years)				699	385	55.0

**Table 7:** Scores of the BSID-II (Mean ± SD *) at 18 months of age (*n* = 1016).

BSID-II Scores	Urban Area (*n* = 416)	Coastal Area (*n* = 600)	*p-*Value **
	Mean ± SD *	Mean ± SD *	
MDI ***	89.8 ± 11.9	86.9 ± 10.6	<0.001
PDI ***	84.6 ± 10.6	84.4 ± 10.6	0.793

* SD, standard deviation. ** Student *t*-test. *** MDI, mental developmental index; PDI, psychomotor developmental index.

**Table 8:** Relations of cord-blood THg * and possible confounders to the scores of BSID-II: Standardized regression coefficients (*β*) of multiple regression analysis (*n* = 1016).

Major Independent Variables	MDI **		PDI **	
	*β*	*p*-Value	*β*	*p*-Value
Cord-blood THg *	−0.028	0.380	−0.053	0.104
Child gender	−0.230	<0.001	−0.111	<0.001
Birth weight	0.034	0.261	−0.012	0.696
Birth order	−0.031	0.312	0.049	0.113
Drinking habit during pregnancy	0.035	0.253	−0.044	0.164
Smoking habit during pregnancy	0.000	0.997	0.040	0.197
Raven score ***	0.036	0.238	0.063	0.044
EES score at 18 months ***	0.134	<0.001	0.071	0.025
Contribution rate, *R*^2^	0.101	<0.001	0.080	<0.001

Other independent variables: Testers of the BSID-II and research area. Cord-blood THg was logarithmically transformed. * THg, total mercury. ** MDI, mental developmental index; PDI, psychomotor developmental index. *** Raven, Raven standard progressive matrices; EES, Evaluation of Environmental Stimulation.

**Table 9:** Relations of cord-blood THg ** and possible confounders to the scores of BSID-II: Standardized regression coefficients (*β*) of multiple regression analysis.

Major Independent Variables	Boys (*n* = 523)				Girls (*n* = 493)			
	MDI *		PDI *		MDI *		PDI *	
	*β*	*p*-Value	*β*	*p*-Value	*β*	*p*-Value	*β*	*p*-Value
Cord-blood THg **	−0.036	0.437	−0.122	0.008	−0.017	0.729	0.024	0.616
Birth weight	0.093	0.036	0.045	0.307	−0.051	0.262	−0.085	0.057
Birth order	0.007	0.873	0.026	0.554	−0.085	0.061	0.066	0.142
Drinking habit during pregnancy	−0.021	0.639	−0.039	0.379	0.085	0.062	−0.053	0.236
Smoking habit during pregnancy	0.000	0.999	0.033	0.440	0.012	0.782	0.057	0.203
Raven score ***	0.052	0.239	0.036	0.407	0.006	0.888	0.090	0.045
EES score at 18 months ***	0.092	0.038	0.086	0.052	0.204	<0.001	0.068	0.137
Contribution rate, *R*^2^	0.058	<0.001	0.078	<0.001	0.061	<0.001	0.082	<0.001

Other independent variables: testers of the BSID-II and research area. Cord-blood THg was logarithmically transformed. * MDI, mental developmental index; PDI, psychomotor developmental index. ** THg, total mercury. *** Raven, Raven standard progressive matrices; EES, Evaluation of Environmental Stimulation.

**Table 10:** Relations of cord-blood THg * and possible confounders to the PDI ** of BSID-II: Standardized regression coefficients (*β*) of multiple regression analysis only in boys.

Major Independent Variables	Urban Area (*n* = 220)		Coastal Area (*n* = 303)	
	*β*	*p*-Value	*β*	*p*-Value
Cord-blood THg *	−0.033	0.606	−0.18	0.002
Birth weight	−0.045	0.477	0.091	0.128
Birth order	−0.081	0.202	0.091	0.124
Drinking habit during pregnancy	−0.058	0.362	−0.044	0.450
Smoking habit during pregnancy	−0.016	0.806	0.062	0.282
Raven score ***	−0.098	0.126	0.124	0.033
EES score at 18 months ***	0.039	0.543	0.116	0.048
Contribution rate, *R*^2^	0.148	<0.001	0.052	0.007

Other independent variables: testers of the BSID-II and research area. Cord-blood THg was logarithmically transformed. * THg, total mercury. ** PDI, psychomotor developmental index. *** Raven, Raven standard progressive matrices; EES, Evaluation of Environmental Stimulation.
